# CoNi Alloys Encapsulated in N-Doped Carbon Nanotubes for Stabilizing Oxygen Electrocatalysis in Zinc–Air Battery

**DOI:** 10.3390/nano13111788

**Published:** 2023-06-01

**Authors:** Yao Nie, Xiaoqin Xu, Xinyu Wang, Mingyang Liu, Ting Gao, Bin Liu, Lixin Li, Xin Meng, Peng Gu, Jinlong Zou

**Affiliations:** 1 Key Laboratory of Functional Inorganic Material Chemistry, Ministry of Education of the People’s Republic of China, School of Chemistry and Materials Science, Heilongjiang University, Harbin 150080, China; 18846123539@163.com (Y.N.); xxq18043018732@163.com (X.X.); wangxinyu11222021@163.com (X.W.); lmy_0202@163.com (M.L.); 13204619731@163.com (B.L.); mengx980103@163.com (X.M.); 13208437710@163.com (P.G.); 2 School of Environment and Chemical Engineering, Heilongjiang University of Science and Technology, Harbin 150080, China

**Keywords:** confinement effect, cycle stability, hollow structure, in-situ growth, surface corrosion

## Abstract

Alloy-based catalysts with high corrosion resistance and less self-aggregation are essential for oxygen reduction/evolution reactions (ORR/OER). Here, via an in situ growth strategy, NiCo alloy-inserted nitrogen-doped carbon nanotubes were assembled on a three-dimensional hollow nanosphere (NiCo@NCNTs/HN) using dicyandiamide. NiCo@NCNTs/HN exhibited better ORR activity (half-wave potential (E_1/2_) of 0.87 V) and stability (E_1/2_ shift of only −13 mV after 5000 cycles) than commercial Pt/C. NiCo@NCNTs/HN displayed a lower OER overpotential (330 mV) than RuO_2_ (390 mV). The NiCo@NCNTs/HN-assembled zinc–air battery exhibited high specific-capacity (847.01 mA h g^−1^) and cycling-stability (291 h). Synergies between NiCo alloys and NCNTs facilitated the charge transfer to promote 4e^−^ ORR/OER kinetics. The carbon skeleton inhibited the corrosion of NiCo alloys from surface to subsurface, while inner cavities of CNTs confined particle growth and the aggregation of NiCo alloys to stabilize bifunctional activity. This provides a viable strategy for the design of alloy-based catalysts with confined grain-size and good structural/catalytic stabilities in oxygen electrocatalysis.

## 1. Introduction

Recently, the increasing environmental pollution and the depletion of fossil fuels have prompted the research and development of highly efficient, environmentally friendly, and sustainable energy conversion and storage systems [1,2,3]. As a new energy source in the 21st century [4], rechargeable zinc–air batteries (ZABs) have received great attention [5,6,7]. However, the intrinsically sluggish kinetics of cathodic oxygen reduction and evolution reactions (ORR/OER) are major drawbacks that need to be addressed for ZABs. [2,8]. In alkaline electrolytes, the corresponding procedures of ORR (Equations (1)–(4)) and OER (Equations (5)–(8)) are shown below. For ORR, available redox centers need to be provided to facilitate the chemisorption of O_2_ molecules, the cleavage of O-O bonds, and the dissociation of OH^−^ to further increase the ORR activity, the rate of which in turn depends on the potential-dependent activation energy barrier. The activity of the OER depends mainly on the efficiency of OH^−^ adsorption on the catalyst surface. Catalysts with an abundance of OH^−^ adsorption sites can facilitate this reaction process. Thus, a high overpotential is required to drive ORR/OER.

The following four steps are included in the ORR process:

* + O_2_ + H**^+^** + e**^−^** → *OOH (1)

*OOH + H**^+^** + e**^−^**→ *O + H_2_O (2)

*O + H**^−^** + e**^−^**→ *OH (3)

*OH + H**^−^**+ e**^−^** → * + H_2_O (4)

The following four steps are included in the OER process:

* + OH**^−^** → *-OH + e**^−^** (5)

*-OH + OH**^−^** → *-O + H_2_O + e**^−^** (6)

*-O + OH**^−^** → *-OOH + e**^−^** (7)

*-OOH + OH**^−^** → * + O_2_ + H_2_O + e**^−^** (8)

To date, noble-metal-based materials, such as platinum on carbon (Pt/C) and ruthenium oxide (RuO_2_), are the state-of-the-art electrocatalysts for ORR and OER, respectively [9]. However, it (Pt/C or RuO_2_) still suffers from high cost, lack of bifunctional activity, and poor stability in alkaline electrolytes for ZABs [10]. In recent years, a few non-noble metal catalysts were exploited to replace the noble ones [11,12,13]. Thus, it still holds great promise to rationally design non-precious metal bifunctional electrocatalysts with tunable compositions and catalytic surfaces [2,6,14,15]. 

In recent years, metal-organic frameworks (MOFs) appear to be promising precursors or templates for the construction of alloy-based electrocatalysts, especially for the alloys of transition metals (such as FeCo [16], FeNi [17], CoNi [18]), which have exhibited good electrocatalytic activity, durability, and intermetallic synergy [19]. Metal ions, as the connection points in MOFs with a three-dimensional structure, facilitate the in situ formation of bimetallic alloy-carbon catalysts under thermal treatment [20,21]. The MOF-derived alloy-carbon catalysts usually possess abundant active sites, a porous structure, and high permeability and conductivity, thus facilitating the mass/electron transfer to enhance electrochemical activity [22,23]. In particular, the alloy-carbon catalysts can inhibit the oxidative corrosion of the carbon skeleton to reserve the active sites, thus stabilizing the electrocatalytic activity.

Ding et al. successfully synthesized a type of MOF-derived, three-dimensional carbonaceous matrix that was randomly loaded with numerous FeNi_3_ alloy nanoparticles (NiFe/C). The NiFe/C catalyst exhibited excellent catalytic activity for ORR in 1.0 M KOH electrolyte [24]. Zhang et al. synthesized a novel core-shell architecture consisting of polystyrene cores and Co-based MOF composite shells encapsulated in discrete Fe-based MOF nanocrystallites to prepare the composites composed of CoFe alloy nanoparticles homogeneously distributed in a porous N-doped carbon shell via a thermal treatment [25]. The as-derived CoFe alloy@N-doped carbon exhibited enhanced catalytic activity for ORR [26]. Unfortunately, the electroconductivity of these MOF-derived materials is intrinsically poor because the structure of the derived carbon is usually disordered and amorphous. In addition, high-temperature pyrolysis often leads to the structural collapse of MOFs, which is uncontrollable for the further growth and dispersion of alloy particles. Briefly, the relatively low conductivity and stability restrict the applications of MOF-derived alloy catalysts for electrocatalysis [26,27]. Furthermore, for alloy nanoparticles, at present, it is extremely challenging to suppress their self-aggregation/collapse, enhance the structural stability, and improve the dispersion during construction. Therefore, given the dilemma mentioned above, anchoring or embedding alloy nanoparticles in a highly graphitized carbon matrix should be a feasible strategy to obtain the strongly coupled alloy-carbon nanohybrids with excellent charge transfer ability and substantial chemical stability.

In this study, via an in situ growth strategy, three-dimensional hollow nanospheres, self-assembled with N-doped carbon nanotubes (with inside NiCo alloy nanoparticles at one end of the nanotubes) on the outer surface (NiCo@NCNTs/HN), were achieved by pyrolyzing a mixture of NiCo-MOF and dicyandiamide. The strong coupling between NiCo and the carbon skeleton (including NCNTs and the carbon skeleton of nanospheres) can greatly enhance electrical conductivity, i.e., charge transfer efficiency. Structurally, this three-dimensional hollow carbon matrix modified by one-dimensional hollow nanotubes with high surface area has the ability to expose more active sites to enhance its electrochemical activity. Compositionally, coordinated interactions between the transition metal alloy nanoparticles can regulate the activity. NiCo@NCNTs/HN, with a porous structure, can eagerly enlarge the working area, the conductive networks, the unimpeded electrolyte/gas diffusion pathways, and the interfacial region to well react with electrolytes and reactants. NiCo, with a small amount embedded in the carbon skeleton, can notably relieve the easy accumulation or aggregation caused by the strong interactions. In addition, the NiCo@NCNTs/HN alloy system is able to inhibit some of the decomposition pathways of oxygen-catalyzed intermediates during ORR/OER, and is usually highly tolerant to electrolyte impurities, thus enhancing catalytic stability in the long-term operation. The hollow structure also provides a relatively enclosed space to exert a confinement effect for the overflow of reactants. As expected, the NiCo@NCNTs/HN catalyst exhibited better catalytic activity and stability than commercial Pt/C (ORR) and RuO_2_ (OER) catalysts. This study demonstrates the great potential of MOF-derived bifunctional alloy-electrocatalysts for rechargeable ZABs. 

## 2. Materials and Methods

### 2.1. Preparation of NiCo-MOF

The NiCo-MOF precursor was synthesized by using a previously reported solvothermal method with a slight modification [28]. Here, 39.09 mg of Ni(NO_3_)_2_·6H_2_O, 390.09 mg of Co(NO_3_)_2_·6H_2_O, 150 mg of 1,3,5-benzenetricarboxylic acid (H_3_BTC), and 1 g of PVP(MW 58,000) were dissolved in 30 mL of N,N-Dimethylformamide (DMF) solution. The above mixture was stirred vigorously until a pink transparent solution was obtained at room temperature. The clear solution was then transferred to a Teflon-lined stainless-steel autoclave (100 mL) and heated to 150 °C for 6 h. After the stainless-steel autoclave cooled down to room temperature, the obtained red sandalwood precipitate was collected (washed) by centrifugation with DMF (three times) and ethanol (three times) orderly. The purplish red solid was dried in a vacuum oven at 60 °C for 12 h. 

### 2.2. Synthesis of Co-MOF and Ni-MOF 

Co-MOF and Ni-MOF were prepared as monometallic catalyst precursors in order to compare their electrocatalytic performance with bimetallic alloy catalysts. The synthesis process of Co-MOF was similar to that of NiCo-MOF, except for the removal of Ni(NO_3_)_2_·6H_2_O during the synthesis, and the mass of Co(NO_3_)_2_·6H_2_O was adjusted to 430 mg. For the preparation of Ni-MOF, 1.279 g of Ni(NO_3_)_2_·6H_2_O and 0.504 g of H_3_BTC were completely dissolved in 70 mL of methanol by stirring for 1 h at room temperature. Then, it was transferred to a 100 mL Teflon-lined autoclave and kept at 150 °C for 6 h. The obtained sample was collected (washed) by centrifugation with DMF (three times) and ethanol (three times) orderly. Finally, the light green materials (Ni-MOF) were obtained by drying in a vacuum oven for 12 h at 60 °C. 

### 2.3. Preparation of NiCo@NCNTs/HN, NiCo/HN, Co@NCNTs/HN, and Ni@NCNTs/HN

**Scheme 1:**
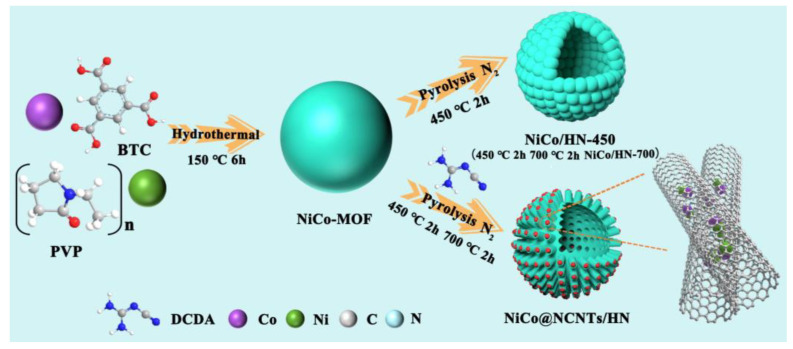
Synthesis route for NiCo/HN-450, NiCo/HN-700, and NiCo@NCNTs/HN catalysts.

In this procedure, 35 mg of NiCo-MOF and 0.7 g of DCDA were placed in two separate porcelain boats in a tube furnace. In particular, DCDA was located upstream of the tube furnace. The above two porcelain boats were firstly heated to 450 °C and maintained at 450 °C for 2 h, and then they were raised to 700 °C and maintained at 700 °C for 2 h under a N_2_ atmosphere at a heating rate of 5 °C min^−1^ to gain the black NiCo@NCNTs/HN sample (without DCDA, the obtained sample was marked as NiCo/HN-700). In addition, the porcelain boats were also heated to 450 °C and maintained at 450 °C for 2 h to obtain the sample named NiCo@NCNTs/HN-450. Co@NCNTs/HN or Ni@NCNTs/HN was synthesized using Co-MOF or Ni-MOF as the precursor, respectively, via a similar procedure to that of NiCo@NCNTs/HN. The flow chart of sample preparation is shown in Scheme 1. In this study, commercial Pt/C (10 wt.%) and RuO_2_ were used as the reference catalysts for ORR and OER, respectively. Detailed methods and instruments for material characterizations and electrochemical tests are described in the ‘Supporting Information’.

## 3. Results

### 3.1. Structural and Compositional Analyses

**Figure 1:**
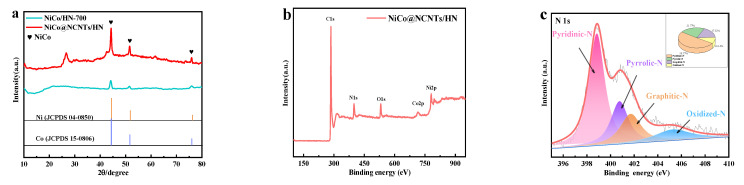

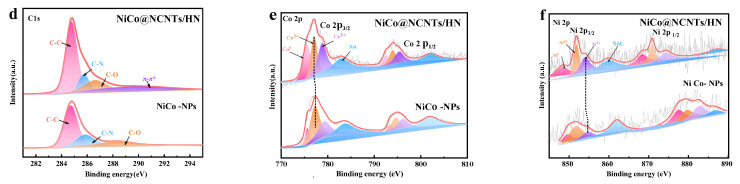
XRD patterns of NiCo/HN-700 and NiCo@NCNTs/HN (**a**); XPS survey spectrum (**b**); high-resolution XPS spectra for N 1s (**c**), C 1s (**d**), Co 2p (**e**), and Ni 2p (**f**) for NiCo@NCNTs/HN.

In Figure 1a, the crystalline structures and phases of the samples are verified using the typical powder X-ray diffraction (XRD) pattern. The peaks located at around 44.2°, 51.6°, and 75.9° were ascribed to the (1 1 1), (2 0 0), and (2 2 0) crystal planes of the NiCo alloy (JCPDS, No. 15-0806) (JCPDS, No. 04-0850), respectively [20,29]. Furthermore, compared with pure NiCo/HN-700, the relatively broad peak at around 26.7° of NiCo@NCNTs/HN was attributed to the (002) crystal plane of graphite carbon. Thus, it clearly demonstrated that with DCDA, the high graphitization degree of carbon skeletons can be obtained due to the formation of N-doped CNTs. Generally, a high degree of graphitization can improve the conductivity of the catalyst, which is beneficial to the charge transfer during the electrolysis process [30]. The XRD patterns of Ni@NCNTs/HN and Co@NCNTs/HN are shown in Figure S1. The diffraction peaks correspond to pure metallic Co^0^ (JCPDS, No. 15-0806) or Ni^0^ (JCPDS, No. 04-0850), which further shows that Co^0^ or Ni^0^ is successfully formed in the structure of NCNTs/HN. The (002) crystal plane of graphite carbon was also observed for Ni@NCNTs/HN or Co@NCNTs/HN.

The chemical status and elemental compositions of NiCo@NCNTs/HN were analyzed using x-ray photoelectron spectroscopy (XPS). Figure 1b shows that C, N, O, Ni, and Co existed on the surface of NiCo@NCNTs/HN. Table S1 shows the proportions of various elements in the catalysts. In Figure 1c, the high-resolution N1s spectrum can be deconvolved into four components at around 398.8, 400.7, 401.7, and 405.2 eV, which were assigned to pyridinic N (50.77%), pyrrolic N (21.77%), graphitic N (17.32%), and oxidized N (10.14%), respectively. N doping can cause the arrangement of the C matrix, promote the accumulation of positive charges, and change the electronic structure of the catalyst, improving the catalytic activity of the catalyst [31]. Graphitic N can improve the conductivity of carbon networks to promote the limiting current density [32]. Due to the electron-donating property of pyridinic-N, it is considered an effective active site for ORR [33]. Pyrrolic N can improve ORR activity by promoting oxygen capture and O-O bond cleavage [34]. The mild oxidation of pyridine-N leads to the formation of oxidized-N [35]. In Figure 1d, the C1s spectrum is decomposed into four peaks at around 284.7, 285.7, 286.6, and 290.1 eV, which corresponded to C-C, C-N, C-O, and π-π*, respectively, confirming the successful N-doping into the CNTs framework [36].

In the Co 2p spectra (Figure 1e), there were four pairs of peaks, including Co^0^ (779.0/794.3 eV), Co^3+^ (780.4/795.6 eV), Co^2+^ (782.0/796.9 eV), and shakeup satellite peaks (785.1/800.1 eV) [36,37]. Likewise, four peaks also appeared in the Ni 2p XPS spectra in Figure 1f, which were assigned to Ni^0^ (851.2 and 869.9 eV), Ni^2+^ (854.3 and 872.1 eV), and Ni^3+^ (856.5 and 875.5 eV) [36,38]. The peak positions of NiCo@NCNTs/HN negatively shifted by comparing with pure NiCo/HN-700, which may be attributed to the electron transfer between NiCo alloy and NCNTs [15]. The formation of the NiCo alloy was confirmed by the presence of metallic Co^0^ and Ni^0^ species, which is well in agreement with the XRD results. The formation of Co^2+^ and Ni^2+^ is due to the oxidation of the outer surface of the NiCo alloy. It is worth noting that the existing Co^3+^ and Ni^3+^ components are the main sources of active species for OER [39]. Meanwhile, the intensity of Co^3+^ (780.4 eV) accounts for a higher part than that of Co^2+^ (782.0 eV), which is in favor of water oxidation to enhance OER activity [40]. In addition, Figure S2 shows the presence of O species on the surface of NiCo@NCNTs/HN and NiCo/HN-700.

**Figure 2:**
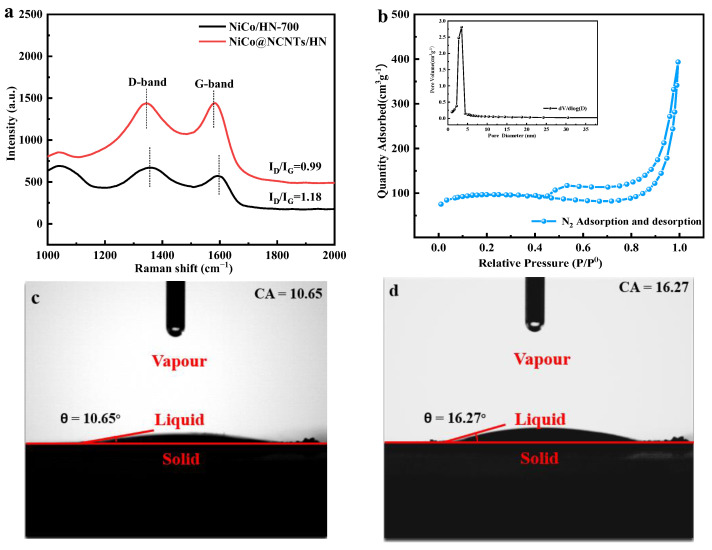
Raman spectra (**a**); N_2_ adsorption/desorption isotherm and pore size distribution (inset) for NiCo@NCNTs/HN (**b**); contact angles of NiCo@NCNTs/HN (**c**) and NiCo/HN-700 (**d**).

Raman tests were used to investigate the structural defects and degree of graphitization of the catalysts (Figure 2a). The D-band (around 1350 cm^−1^) is related to the topological defects and disorder degree of carbonaceous materials, while the G-band (around 1580 cm^−1^) represents the in-plane stretching vibration of the hybridization of C atoms. The smaller the I_D_/I_G_ ratio, the higher the degree of graphitization of carbon [41,42]. In Figure 2a, the I_D_/I_G_ values of NiCo@NCNTs/HN and NiCo/HN-700 are 0.99 and 1.18, respectively, implying that a higher degree of graphitization was obtained by NiCo@NCNTs/HN. It revealed that the NiCo alloys encapsulated by CNTs may lead to the formation of disordered edge structure and defects in the carbon skeleton [43]. These defects in the carbon structure can generate the electrocatalytically efficient active sites. Figure 2b and Figure S3a,b show the N_2_ adsorption and desorption isotherm, as well as the pore size distribution curves of NiCo@NCNTs/HN, Ni@NCNTs/HN, and Co@NCNTs/HN, respectively. There was a typical IV-type hysteresis under high relative pressures, implicating that they mainly consist of a mesoporous structure. The specific surface areas of Ni@NCNTs/HN, Co@NCNTs/HN, and NiCo NCNTs/HN are 226, 235, and 240 m^2^ g^−1^ (Tables S2–S4), respectively, while the pore size distribution curve further confirms the mesoporous character. The pores can provide sufficient active centers for absorbing, transforming, and desorbing the reactants/intermediates during oxygen electrocatalysis [44]. They also reveal that NiCo@NCNTs/HN possesses typical mesopores to allow the efficient permeation of the electrolyte, so as to facilitate mass transfer [44,45]. Figure 2c,d shows that NiCo@NCNTs/HN exhibited a smaller contact angle (10.65°) than NiCo/HN-700 (16.27°). This means that NiCo@NCNTs/HN had a more hydrophilic surface [46]. Because ORR is a three-phase interfacial reaction, the catalyst with strong hydrophilicity can promote the interaction between the electrolyte and catalyst [47]. Oxygen molecules first need to be physically adsorbed on the catalyst (in contact with the catalyst) and then activated on the active site. The deep wetting helps electrolytes to enter the pores and increases the contact probability between active sites and reactants, thereby promoting electrocatalytic efficiency [48].

**Figure 3:**
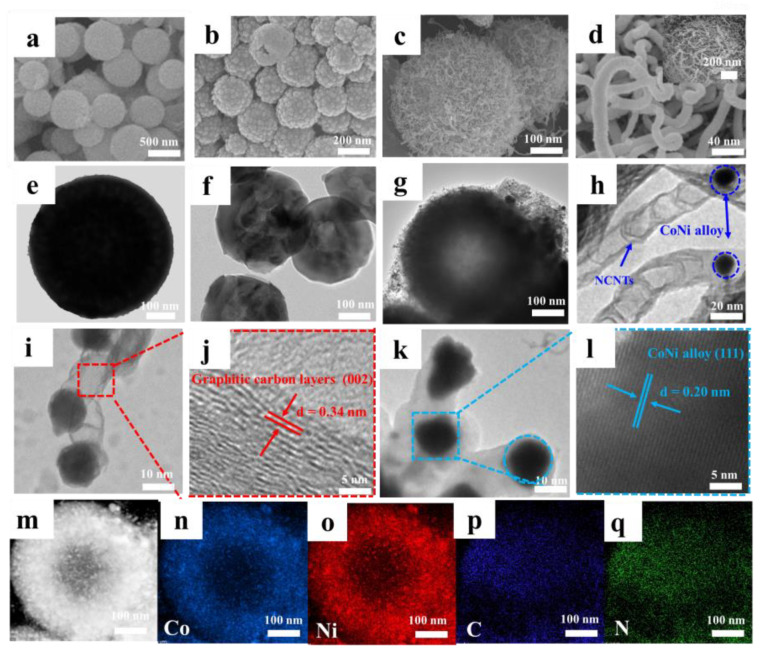
SEM images of NiCo-MOF (**a**), NiCo/HN-450 (**b**), and NiCo@NCNTs/HN (**c**,**d**); TEM images of NiCo-MOF (**e**), NiCo@C-450 (**f**), and NiCo@NCNTs/HN (**g**,**h**); HRTEM images of NiCo@NCNTs/HN (**i**–**l**); HAADF-STEM image of NiCo@NCNTs/HN (**m**); element mapping of Co (**n**), Ni (**o**), C (**p**), and N (**q**).

In Figure 3a, the SEM image of NiCo-MOF shows that H_3_BTC and metal ion underwent a coordination reaction at 150 °C to form a spherical structure by the simple hydrothermal method. Obviously, the spheres had an average size of 500 nm and were monodispersed, with a uniform morphology and smooth surface. Figure S4 shows that the SEM images of Co-MOF and Ni-MOF are the same as that of NiCo-MOF. Figure 3b shows that the surface of NiCo-MOF spheres annealed at 450 °C exhibited a rather rough surface consisting of small nanoparticles. The average size of a NiCo/HN-450 particle (without DCDA) is approximately 200 nm, indicating that the skeleton shrinkage of the sphere clearly occurred. After carbonization at 700 °C, NCNTs derived from the pyrolysis of DCDA were successfully planted on the surface of the nanosphere, and the NiCo-MOF-derived NiCo alloy nanoparticles were fully encapsulated in the nanotubes (Figure 3c,d). Normally, this unique wool-ball-like structure should not only supply a large contact area between active sites and electrolytes, but it should also effectively reduce the self-aggregation of NiCo alloys inside the wool (NCNTs).

In Figure 3e, it shows that the NiCo-MOF precursor was a solid sphere (TEM image). NiCo/HN-450 showed a spherical non-solid structure with many pores (Figure 3f). In Figure 3g, a hollow spherical structure, coated with NCNTs with an average diameter of approximately 20 nm derived from the pyrolysis of DCDA, is observed. In Figure 3h, it shows that the NCNTs had a bamboo-like structure, with NiCo nanoparticles encapsulated in one end of the nanotubes. This structure with a bimetallic alloy core inside and a graphite shell outside facilitated the contact between the active sites and the electrolyte, and it prevents the erosion and aggregation of CoNi alloy NPs in the electrolyte [49]. In Figure 3i,j, the HRTEM image of NiCo@NCNTs/HN reveal that the NiCo alloys were tightly embedded into the graphitized carbon with an interlayer spacing of ≈0.34 nm, which corresponds to the (002) plane of NCNTs. The lattice fringe spacing of 0.20 nm (Figure 3k,l) can be assigned to the (111) crystal plane of the CoNi alloy. These structural features of the catalyst can maintain high electrocatalytic activity and long-term stability. In Figure 3m–q, the high-angle annular dark field (HAADF)-STEM element mapping images demonstrate that C, N, Ni, and Co were homogeneously dispersed in NiCo@NCNTs/HN. The visible bright spots in different colors indicated the presence and distribution of each element. It eagerly revealed the successful formation of the NiCo alloy, the introduction of N doping, and the perfect realization of the hollow structure. 

### 3.2. Electrocatalytic Activities of NiCo@NCNTs/HN Catalyst for ORR 

**Figure 4:**
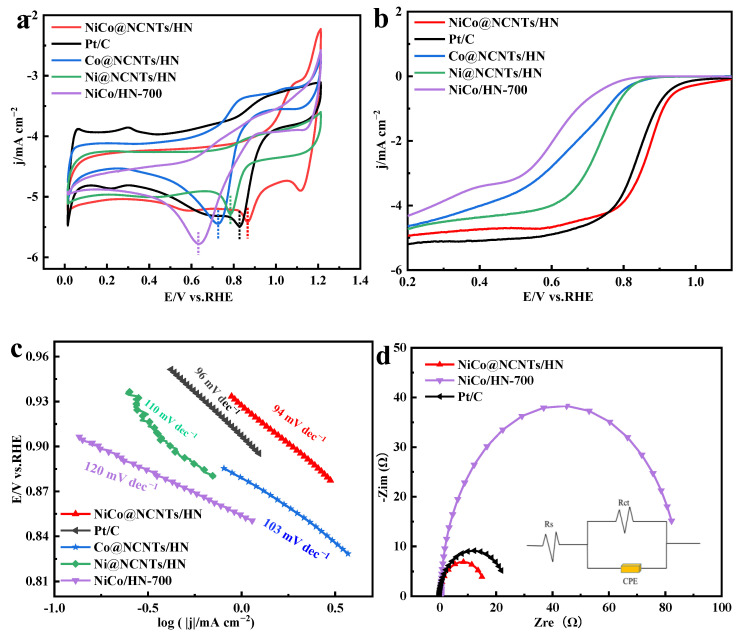

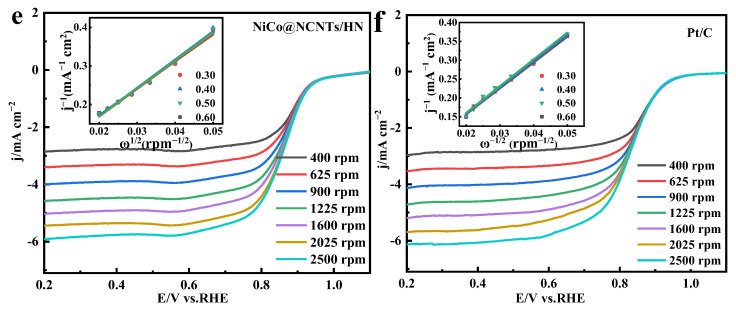
CV curves for NiCo@NCNTs/HN, Co@NCNTs/HN, Ni@NCNTs/HN, NiCo/HN-700, and Pt/C in an O_2_-saturated 0.1 M KOH solution at a scan rate of 10 mV s^−1^ (**a**); LSV curves for NiCo@NCNTs/HN, Co@NCNTs/HN, Ni@NCNTs/HN, NiCo/HN-700, and Pt/C (5 mV s^−1^ and 1600 rpm) in an O_2-_saturated 0.1 M KOH solution (**b**); Tafel plots of NiCo@NCNTs/HN and Pt/C (**c**); Nyquist curves of NiCo@NCNTs/HN and Pt/C in an O_2_-saturated 0.1 M KOH solution (an amplitude of 5 mV and 1600 rpm) (**d**); LSV curves and calculated K-L plots (insets) at different potentials (5 mV s^−1^) for NiCo@NCNTs/HN (**e**) and Pt/C (**f**).

To initially evaluate the ORR activities of Pt/C, Ni@NCNTs/HN, Co@NCNTs/HN, NiCo/HN-700, and NiCo@NCNTs/HN catalysts, cyclic voltammetry (CV) tests were performed in a 0.1 M O_2_-saturated KOH solution [50]. In Figure 4a, the reduction peak of the NiCo@NCNTs/HN was located at around 0.87 V, which was superior to those of Pt/C (0.83 V), Ni@NCNTs/HN (0.78 V), Co@NCNTs/HN (0.72 V), and NiCo/HN-700 (0.63 V). As reported previously, the inevitable oxidation of CoNi alloys in the hybrids can form the active Co(II) species, which is directly responsible for the high ORR activity [51,52,53,54,55]. Co(II) can change the chemisorption mode of oxo intermediates due to the strong interaction between the orbital of the oxygen molecule and the d orbital of central atomic vacancy, thus effectively weakening the O-O bond to facilitate the dissociation of oxygen molecules to produce H_2_O [51,52]. It is noteworthy that, compared to NiCo/HN-700, the excellent ORR activity of the NiCo@NCNTs/HN catalyst was chiefly attributed to the introduction of N-doped CNTs, with high electrical conductivity, and the N-species served as highly active sites for ORR [20,56]. Moreover, the self-grown NCNTs on the surface of the hollow spherical structure can promote the charge transfer between CoNi alloys and NCNTs to gain the excellent ORR activity.

The ORR activities of various catalysts were further investigated by linear sweep voltammetry (LSV) tests [5]. In Figure 4b, the half-wave potential (E_1/2_, 0.877 V) and onset potential (E_onset_, 0.92 V) of NiCo@NCNTs/HN were higher than those of commercial Pt/C (E_1/2_ = 0.835 V and E_onset_ = 0.91 V), NiCo/HN-700 (E_1/2_ = 0.64 V and E_onset_ = 0.72 V), Ni@NCNTs/HN (E_1/2_ = 0.73 V and E_onset_ = 0.79 V), and Co@NCNTs/HN (E_1/2_ = 0.72 V and E_onset_ = 0.78 V). In Table S5, the electrocatalytic performance of NiCo@NCNTs/HN is also comparable to many of the most advanced non-precious metal catalysts that have been recently reported. Importantly, the large specific surface area of the hollow structure makes multiple active sites meaningful, and O_2_ bubbles and electrolytes can easily access the active sites and facilitate the gas diffusion and mass transfer, which may be the main reason for the high diffusion current density of NiCo@NCNTs/HN [57]. In addition, the synergistic effects between NiCo alloys and NCNTs can greatly increase the electrical conductivity (charge transfer) and further accelerate the ORR process [58,59]. To evaluate the ORR kinetics of the NiCo@NCNTs/HN catalyst, we constructed Tafel plots based on the LSV curves of several catalysts at 1600 rpm (Figure 4c). NiCo@NCNTs/HN (94 mV dec^−1^) showed a smaller Tafel slope than Pt/C (96 mV dec^−1^), Co@NCNTs/HN (103 mV dec^−1^), Ni@NCNTs/HN (110 mV dec^−1^), and NiCo/HN-700 (120 mV dec^−1^). In general, the smaller the Tafel slope, the higher the catalytic activity [60,61]. In Figure 4d and Figure S5a, the charge transfer resistance (R_ct_) values for NiCo@NCNTs/HN, NiCo/HN-700, and Pt/C are 15.88, 82.44, and 23.60 Ω, respectively. The lower the R_ct_, the higher the charge transfer rate [2]. This indicates that NiCo@NCNTs/HN has an efficient charge transfer capability and favors the effective ORR process, which is consistent with the Tafel results. The lower resistance should be ascribed to the increased conductivity due to the formation/coating of the N-doped CNTs [57]. In Figure 4e,f, RDE tests were performed at different rotation speeds (400 to 2025 rpm) to evaluate the ORR diffusion kinetics of NiCo@NCNTs/HN and Pt/C. The electron transfer number (n) can be obtained by calculating the slope of the linear plot obtained using Koutecky–Levich (K–L) curves at different potentials [62]. At different potentials from 0.30 to 0.60 V, it was clearly seen that the K–L plots of NiCo@NCNTs/HN and Pt/C showed a linear relationship and almost overlapped each other, indicating that the ORR process on the NiCo@NCNTs/HN catalyst is close to the 4e^−^ reaction pathway.

**Figure 5:**
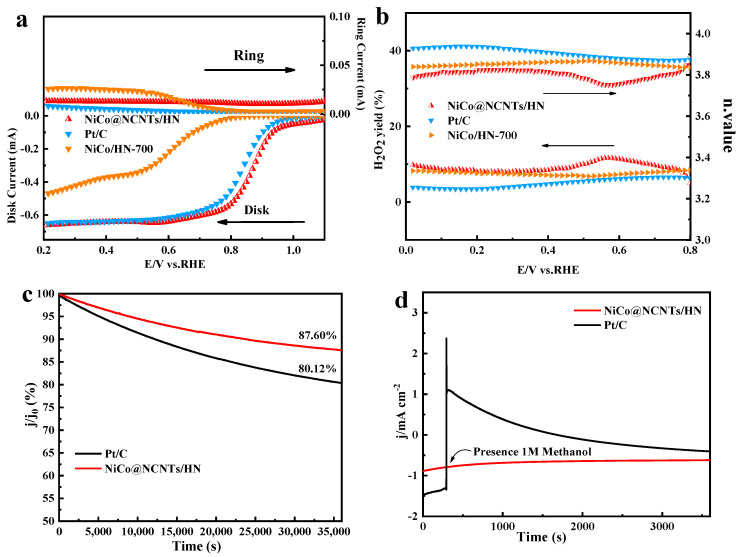
RRDE voltammograms of Pt/C, NiCo/HN-700, and NiCo@NCNTs/HN in an O_2_-saturated 0.1 M KOH solution (**a**); H_2_O_2_ yields and the corresponding electron transfer number (n) (**b**); chronoamperometric (i-t) responses of NiCo@NCNTs/HN and Pt/C before (**c**) and after (**d**) the addition of 3 mL of methanol (1.0 M) in an O_2_-saturated 0.1 M KOH at 1600 rpm.

In Figure 5a,b, RRDE tests were performed to determine H_2_O_2_ yields and n values at different potentials in alkaline media. The H_2_O_2_ yields of NiCo@NCNTs/HN, NiCo/HN-700, and Pt/C were in the range of 5.21 to 9.89%, while the n values were in the range of 3.89 to 3.97 (Figure S6). It was revealed that the NiCo@NCNTs/HN catalyst exhibited high selectivity and activity for the conversion of O_2_ to OH^−^ via a 4e^−^ ORR pathway, which is consistent with the RDE results. Superior electrocatalysts not only require high catalytic activity, but they also need promising stability to assess their application feasibility. In Figure 5c, the NiCo@NCNTs/HN catalyst maintained 87.60% of the initial current density after 36,000 s (10 h), while the Pt/C electrode maintained 80.12% of initial current density, indicating that the ORR stability of NiCo@NCNTs/HN is quite ideal. The catalytic stability of the catalysts was further evaluated by the methanol tolerance test. In Figure 5d, after the addition of 3 mL of methanol, the current density of Pt/C jumped sharply at 300 s, because the methanol oxidation reaction or the CO toxicity to the active sites caused a significant loss of ORR activity [19]. In contrast, no significant current change was observed for NiCo@NCNTs/HN, indicating that it has a good methanol tolerance for ORR. The accelerated durability test (ADT) revealed that, after 5000 cycles, E_1/2_ had a negative shift of only 13 mV (Figure S7a) for NiCo@NCNTs/HN, which was much smaller than that of Pt/C (20 mV) (Figure S7b). The high stability is ascribed to the NiCo alloys being tightly surrounded by a carbon wall to effectively protect the bimetallic alloys from dissolution and oxidation, and the size of the wrapped alloy particles can be effectively controlled to eagerly prevent particle aggregation [55,63].

### 3.3. OER Performance on NiCo@NCNTs/HN Catalyst

**Figure 6:**
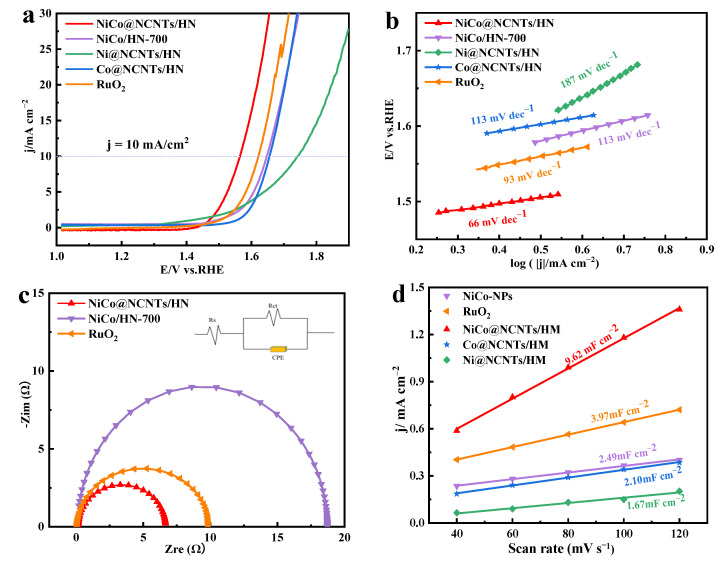
LSV curves (2 mV s^−1^ and 1600 rpm) (**a**) and Tafel plots (**b**) of RuO_2_, NiCo@NCNTs/HN, Co@NCNTs/HN, Ni@NCNTs/HN, and NiCo/HN-700; Nyquist curves of NiCo@NCNTs/HN, NiCo/HN-700, and RuO_2_ at an amplitude of 5 mV ((**c**), 1600 rpm); linear plots of scan rates vs. current density for NiCo@NCNTs/HN, NiCo/HN-700, Co@NCNTs/HN, Ni@NCNTs/HN, and RuO_2_ in 0.1 M KOH electrolyte (**d**).

As shown in Figure 6a, at 10 mA cm^−2^, NiCo@NCNTs/HN obtained the lowest overpotential in driving the catalytic reaction (330 mV) compared to RuO_2_ (390 mV), NiCo/HN-700 (410 mV), Co@NCNTs/HN (420 mV), and Ni@NCNTs/HN (510 mV). Notably, the synergistic interactions between CoNi alloys and NCNTs contributed to the generation of a sufficient number of viable catalytically active species (CoOOH/NiOOH). As reported previously, the special interfacial structure of the NiCo alloy can enhance the OER catalytic activity by regulating the in situ formation of oxyhydroxides (NiOOH/CoOOH) on the surface of the alloy [64,65]. As shown in Table S6, NiCo@NCNTs/HN also exhibited excellent OER activity compared to the recently reported OER performance of non-precious metal electrocatalysts [63]. In Figure 6b, the Tafel slope of NiCo@NCNTs/HN was 66 mV dec^−1^, which was significantly better than those of NiCo/HN-700 (113 mV dec^−1^), Co@NCNTs/HN (113 mV dec^−1^), Ni@NCNTs/HN (187 mV dec^−1^), and RuO_2_ (93 mV dec^−1^). The low slope revealed that the NiCo@NCNTs/HN catalyst is more favorable for OER, which can be recognized as having a high potential for commercial application [66,67].

In Figure 6c (EIS curve), NiCo@NCNTs/HN had the lowest R_ct_ (6.5 Ω) among the catalysts of RuO_2_ (9.8 Ω), NiCo/HN-700 (19 Ω), and other catalysts (Figure S5b). This suggests that the charge transfer between NiCo alloys and NCNTs can be conducted rapidly to obtain super OER kinetics [57]. Double-layer capacitance (C_dl_) was employed to evaluate the electrochemical surface area (ECSA) of catalysts. CV tests at different scan rates were used to obtain the C_dl_ and ECSA (Figure S8). In Figure 6d, the C_dl_ values for NiCo@NCNTs/HN, NiCo/HN-700, Co@NCNTs/HN, Ni@NCNTs/HN, and RuO_2_ were 9.62, 2.49, 2.10, 1.67, and 3.97 mF cm^−2^, respectively, indicating that more active species (CoOOH/NiOOH) should be generated on the surface of NiCo@NCNTs/HN [68]. Accordingly, the ECSA values of NiCo@NCNTs/HN, NiCo/HN-700, Co@NCNTs/HN, Ni@NCNTs/HN, and RuO_2_ were 240.5, 62.25, 52.5, 41.75, and 99.25 cm^2^, respectively.

**Figure 7:**
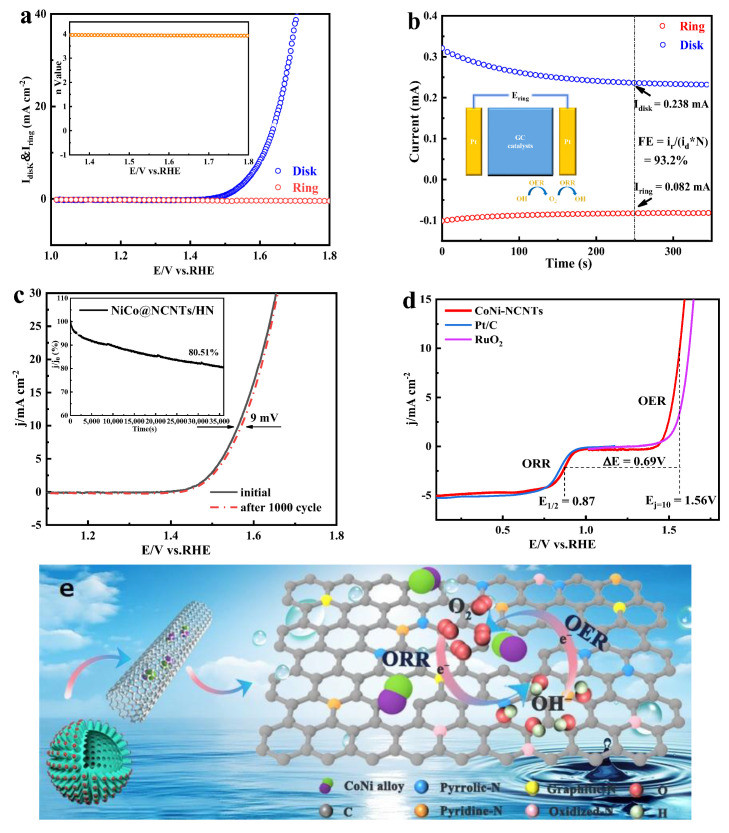
Voltammogram for NiCo@NCNTs/HN in 0.1 M KOH on an RRDE (1600 rpm) device, and the inset shows the corresponding electron transfer number (n) as a function of applied potentials (**a**); the faradaic efficiency measurement of NiCo@NCNTs/HN on an RRDE (1600 rpm) device (**b**); OER polarization curves of NiCo@NCNTs/HN before and after the continuous operations (**c**) (inset shows the i-t responses in 0.1 M KOH at 1600 rpm); polarization curves of NiCo@NCNTs/HN and Pt/C (or RuO_2_) catalysts using the three-electrode system in the full OER/ORR region (**d**); possible ORR/OER mechanisms of the NiCo@NCNTs/HN catalyst (**e**).

In Figure 7a and inset (RRDE tests), an average n value of 3.90 was obtained, indicating that the OER process on the NiCo@NCNTs/HN proceeded through the 4e^−^ pathway (4OH^−^→ 2H_2_O + O_2_ + 4e^−^). The Faraday efficiency of NiCo@NCNTs/HN was measured by using RRDE. In Figure 7b, when a current of 0.238 mA was applied to the disc electrode to produce O_2_, a ring current of 0.082 mA was detected, and the Faraday efficiency was 93.2%. In Figure 7c, after 1000 LSV cycles, the E_j=10_ of NiCo@NCNTs/HN increased by only 9 mV in the potential range of 1.0–1.8 V (vs. RHE). As shown in the inset of Figure 7c (chronoamperometry), the excellent OER stability of NiCo@NCNTs/HN was chiefly related to the protective effect of the carbon skeleton for wrapping the NiCo alloys. Moreover, the high mechanical stability of the carbon skeleton can prevent the corrosion of NiCo oxyhydroxides, thus enhancing the catalytic durability to OER in alkaline electrolytes. To verify the origin of the excellent catalytic performance of the NiCo@NCNTs/HN catalyst in terms of OER, in situ XRD tests were performed (Figure S9). During OER, several new diffraction peaks appeared at around 36.27° and 63.96°, corresponding to the (1 0 1) and (0 0 2) planes of β-NiOOH (JCPDS 27-0956) [46], respectively. Moreover, new diffraction peaks at around 41.40° and 45.79° should correspond to the (0 0 6) and (1 0 4) planes of γ-CoOOH (JCPDS No. 07-0169) [15], respectively. Thus, this confirms that NiOOH/CoOOH derived from Ni^0^/Co^0^ (NiCo alloy) was the actual active component for OER. XRD and SEM techniques were used to investigate the structural stability of NiCo@NCNTs/HN after the stability tests. As shown in Figure S10a, the XRD pattern did not change significantly, and the crystalline structure of the pure alloy was also maintained after the test, which confirms the good stability of the NiCo@NCNTs/HN catalyst. As can be seen in Figure 3c and Figure S10b, the surface of NiCo@NCNTs/HN became slightly rougher after up to 10 h of tests, but the overall structure remained unchanged. Moreover, it was also revealed that the attached particles were homogeneously distributed and only slightly aggregated on the surface [69]. The potential difference between OER and ORR (ΔE = E_j10_^OER^ − E_1/2_^ORR^) was a useful indicator for evaluating the bifunctional activity of the catalyst. A smaller value of ΔE implies higher overall bifunctional activity [20]. As shown in Figure 7d, with a ΔE value of 0.69 V, the bifunctional activity of the NiCo@NCNTs/HN catalyst was higher than that of Pt/C + RuO_2_ (0.74 V). Compared with the recently reported bifunctional catalysts (Figure S11 and Table S7), NiCo@NCNTs/HN still had a promising ΔE. Therefore, NiCo@NCNTs/HN can be considered a potential bifunctional catalyst for application in ZAB. In Figure 7e, the alloy nanoparticles located inside of the nanotube were closely protected by the external NCNTs to inhibit their rapid degradation and thus improve ORR/OER stabilities. In addition, the hollow space of HN can enhance the mass transfer rate during the electrocatalytic process, thus accelerating the formation/reaction of oxo intermediates (OOH*, O*, and *OH). The NiOOH/CoOOH species on the surface of the NiCo alloy can greatly lower the energy barrier for OER, and these active species can also promote the charge transfer, thus effectively promoting the OER activity.

### 3.4. Application of NiCo@NCNTs/HN Catalyst in ZAB

**Figure 8:**
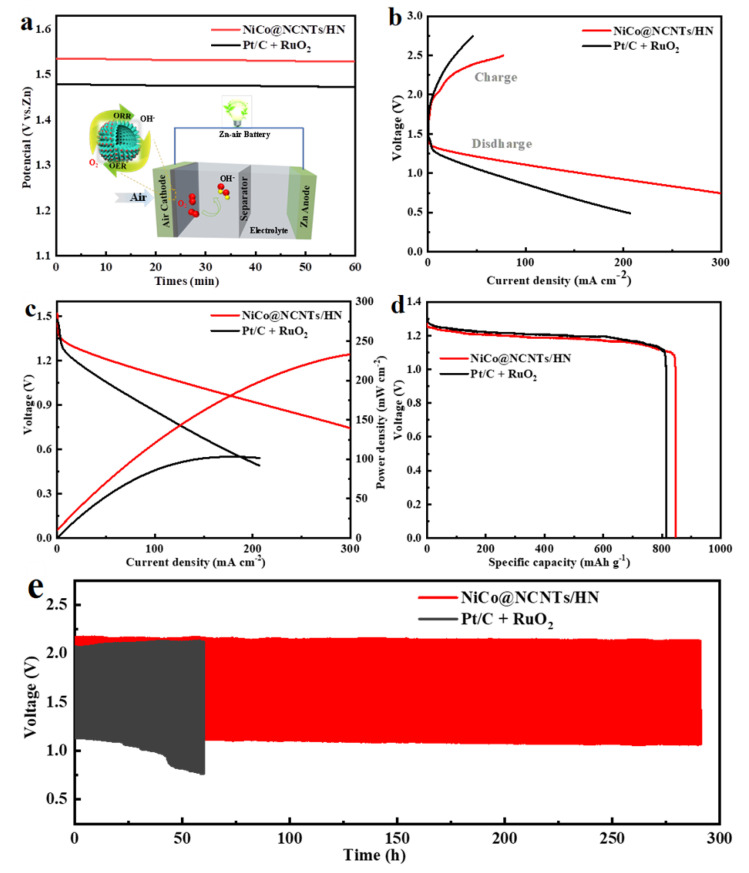
Open-circuit plots of primary ZABs assembled with NiCo@NCNTs/HN and Pt/C + RuO_2_ (**a**); discharging polarization curves and corresponding power density plots (**b**); discharge polarization curves and corresponding power densities of NiCo@NCNTs/HN- and Pt/C + RuO_2_-based ZABs (**c**); discharge curves of ZABs at 10 mA cm^−2^ (**d**); galvanostatic charge–discharge curve of NiCo@NCNTs/HN-based rechargeable ZAB (**e**).

The desirable bifunctional activity and stability of the NiCo@NCNTs/HN catalyst prompted us to evaluate their suitability for ZAB. A rechargeable ZAB was constructed using carbon cloth (~2.5 mg cm^−2^) loaded with NiCo@NCNTs/HN (or Pt/C + RuO_2_) as the air cathode, Zn as the anode, and a mixed solution of 6 M KOH + 0.2 M Zn acetate as the electrolyte (Figure 8a). As shown in Figure 8a, the open circuit voltage (OCP) of NiCo@NCNTs/HN-assembled ZAB was approximately 1.53 V, while the reference ZAB with Pt/C + RuO_2_ was 1.47 V, implying that the as-prepared catalyst had good practicality [70]. Figure 8b shows the charge/discharge polarization curves of the ZABs, and the NiCo@NCNTs/HN cathode presented a relatively lower charge voltage, a higher discharge voltage, and a smaller charge/discharge overpotential gap compared to the reference, Pt/C + RuO_2_. In addition, the maximum power density of the NiCo@NCNTs/HN cathode was 232.68 mW cm^−2^ (Figure 8c), which exceeds that of the commercial Pt/C + RuO_2_ (185.82 mW cm^−2^). This excellent discharge capacity is extremely consistent with the outstanding OER/ORR activities [71,72]. In Figure 8d, the specific discharge capacity of ZAB with NiCo@NCNTs/HN was 847.01 mA h g^−1^, which was superior to that of ZAB with Pt/C + RuO_2_ (813.49 mA h g^−1^). At 10 mA cm^−2^ (Figure 8e), the NiCo@NCNTs/HN cathode possessed a much better cycling stability (291 h) compared to the Pt/C + RuO_2_ cathode (45 h). The tight connection between NiCo alloys and NCNTs created many highly active interfaces to stabilize the long-term OER/ORR activities. The inner cavities of CNTs provide a perfect space to confine NiCo alloys to prevent the aggregation and leaching of the NiCo alloys, thus greatly improving long-term electrocatalytic stability [73,74]. This highlights the long-term stability and high efficiency of NiCo@NCNTs/HN-assembled ZAB.

## 4. Conclusions

In summary, a novel 3D electrocatalyst with surface-grown NCNTs, a large surface area, and a hollow structure was successfully prepared using a hydrothermal-annealing strategy. The skeleton of the sphere provided ideal platforms for the uniform and dense growth of bamboo-like CNTs to encapsulate the NiCo alloy nanoparticles. The NiCo@NCNTs/HN catalyst exhibited excellent ORR (E_1/2_ = 0.87 V) and OER (E_j=10_ = 1.56 V) activities in alkaline media. After 36,000 s, the decline of the ORR current density of NiCo@NCNTs/HN was only 12.40%, while after 1000 OER cycles, the E_j=10_ of NiCo@NCNTs/HN increased by only 9 mV. As the air-cathode, the ZAB with the NiCo@NCNTs/HN catalyst provided a high open-circuit voltage (1.53 V) and power density (847.01 mA h g^−1^). The NiCo alloy in situ encapsulated by NCNTs with uniform dispersion and ultrafine size can stabilize the exposure of active sites to the electrolyte, which contributes to the high bifunctional activity (ΔE = 0.69 V) and stability of NiCo@NCNTs/HN. The excellent activity/stability are also ascribed to the efficient charge transfer between NiCo alloys and NCNTs. The controllable construction of this bifunctional electrocatalyst promotes the further development of MOF-derived materials for energy storage and conversion.

## Data Availability

Data can be made available upon request.

## Supplementary Materials

The following supporting information can be downloaded at: https://www.mdpi.com/article/10.3390/nano13111788/s1, Figure S1: XRD patterns of Co@NCNTs/HN (a) and Ni@NCNTs/HN (b); Figure S2: High resolution XPS spectra of O 1s for NiCo@NCNTs/HN (a) and NiCo/HN-700 (b); Figure S3: N_2_ adsorption/desorption isotherm and pore size distribution (inset) for Ni@NCNTs/HN (a) and Co@NCNTs/HN (b); Figure S4: SEM images of Co-MOF (a) and Ni-MOF (b); Figure S5: (a) Nyquist curves of Co@NCNTs/HN and Ni@NCNTs/HN in an O_2_-saturated 0.1 M KOH solution at an amplitude of 5 mV with a rotation rate of 1600 rpm for ORR; (b) Nyquist curves of Co@NCNTs/HN and Ni@NCNTs/HN in an O_2_-saturated 1.0 M KOH solution at an amplitude of 5 mV with a rotation rate of 1600 rpm for OER; Figure S6: Electron-transfer number n derived from K-L plots at different potentials. Figure S7: ORR polarization curves of NiCo@NCNTs/HN (a) and Pt/C (b) before and after the continuous CV tests in the O_2_-saturated 0.1 M KOH at 1600 rpm; Figure S8: CV curves of RuO_2_ (a), NiCo@NCNTs/HN (b), Ni@NCNTs/HN (c), NiCo/HN-700 (d), and Co@NCNTs/HN (e) in 0.1 M KOH electrolyte at different scan rates; Figure S9: In situ XRD patterns of NiCo@NCNTs/HN collected during OER; Figure S10: XRD (a) and SEM (b) of the NiCo@NCNTs/HN sample after the chronopotentiometric test for 10 h; Figure S11: Comparison of OER and ORR activities in this work with representative electrocatalysts in the literature (the dotted lines show the ΔE at constant values). Table S1: Chemical compositions (wt.%) of NiCo@NCNTs/HM electrocatalysts obtained from XPS analyses. Table S2: Textural properties of NiCo@NCNTs/HN. Table S3: Textural properties of Ni@NCNTs/HN. Table S4: Textural properties of Co@NCNTs/HN. Table S5: Summary of ORR activities of various catalysts in alkaline electrolyte. Table S6: Summary of OER activities of various catalysts in alkaline electrolyte. Table S7: Summary of the ORR/OER bifunctional oxygen electrocatalytic activity of various catalysts in alkaline electrolyte. References [14,46,75,76,77,78,79,80,81,82,83,84,85,86,87,88,89,90,91,92,93,94,95,96] are cited in the Supplementary Materials.
