# Supplementary material for: CoNi Alloys Encapsulated in N-Doped Carbon Nanotubes for Stabilizing Oxygen Electrocatalysis in Zinc–Air Battery
Source: Nanomaterials (Basel). 2023 Jun 1;13(11):1788. doi: 10.3390/nano13111788 (PMC10255264; doi:10.3390/nano13111788)
Supplement: Supplementary file 1 [file nanomaterials-13-01788-s001.zip › nanomaterials-2413592-supplementary.pdf]

nanomaterials

Supplementary Materials

CoNi alloys encapsulated in N-doped carbon nanotubes for stabilizing oxygen electrocatalysis in zinc–air battery

Yao Nie [1], Xiaoqin Xu [1], Xinyu Wang [1], Mingyang Liu [1], Ting Gao [1,*], Bin Liu [1], Lixin Li [2,*] Xin Meng [1], Peng gu [1], and Jinlong Zou [1,*]

[1] Key Laboratory of Functional Inorganic Material Chemistry, Ministry of Education of the People's Republic of China, School of Chemistry and Materials Science, Heilongjiang University, Harbin, 150080, China; 18846123539@163.com (Y.N.); xxq18043018732@163.com (X.X.); wangxinyu11222021@163.com (W.Y.); LMY_0202@163.com (L.Y.); 13204619731@163.com (B.L.); mengx980103@163.com (X.M.); 13208437710@163.com (P.G.)

[2] School of Environment and Chemical Engineering, Heilongjiang University of Science and Technology, Harbin 150080, China.

* Correspondence: gaoting@hlju.edu.cn (T. G.); lilixin1980@163.com (L. X); zoujinlong@hlju.edu.cn (J.Z.).

1. Experimental Section

1.1. Material Characterizations

Herein, the structure, morphology and compositions of the as-prepared materials were characterized by x-ray diffraction (XRD), x-ray photoelectron spectroscopy (XPS), N_2 adsorption/desorption isotherms, transmission electron microscopy (TEM), scanning electron microscope (SEM), and elemental mappings (energy dispersive x-ray spectroscopy, EDS). The collection of XRD data was performed using a Rigaku D/max 2500 diffractometer equipped with Cu-Kα radiation (k = 1.5406 Å, 40 kV, 20 mA). The Kratos-AXISUL TRA DLD X-ray photoelectron spectrometer equipped with Al Kα radiation was used to record XPS data. "XPS peak" software was used to fit the XPS data of each element. N_2 adsorption and desorption isotherms were determined at 77 k using a micron-scale three-star adsorption analyzer. The Brunauer-Emmett-Teller (BET) method and Barrett-Joyner-Halenda (BJH) theory were used to analyze the specific surface area and pore size distribution (PSD), respectively. TEM and high-resolution TEM (HRTEM) images were collected on a JEM-2100 electron microscope (JEOL) at an accelerated voltage of 200 kV. SEM images and elemental mappings were collected using an S-4800 (Hitachi, Japan) scanning electron microscope at an acceleration voltage of 5.0 kV. The contact angle of the sample surface was characterized by the JC20001 contact angle tester.

1.2. Electrochemical Measurements

All electrochemical tests were performed on a rotating disk electrode (RDE) controller connected to an electrochemical workstation (CHI760E, Shanghai, Chenhua). A three-electrode system was used for electrochemical tests, with a saturated calomel electrode (SCE) as the reference electrode, a platinum wire as the counter electrode, and a catalyst-coated glassy carbon electrode (GCE) as the working electrode. GC-disc/Pt-ring electrodes (RRDE) were produced by TAI ZHOU KE RUI TE ANALYTICLAL INSTRUMENT Co., Ltd, the disc electrode was coated with the as-prepared catalyst, and the ring electrode was made of platinum. In this study, the measured SCE potential was converted to the reversible hydrogen electrode (RHE) potential based on ERHE = ESCE + 0.0592 pH + 0.2438. The method for preparing the catalyst ink was performed as follows: 5 mg of the catalyst was dispersed in a mixture of 50 μL of 5 wt.% Nafion (Aldrich) and 100 μL ethanol, and then the mixture was sonicated for 30 min to form the uniform ink. The surface of GCE (4 mm in diameter and coating area of 0.126 cm^2) was coated with 5 μL of the prepared ink to achieve a catalyst loading of 1.322 mg cm^{-2}. The area of GCE for RDE

measurements was the same as that of RRDE. Commercial Pt/C (10 wt.%) and RuO_2 were used as reference catalysts in this study.

1.3. Characterization and Electrochemical Test Methods.

Commercial Pt/C (20 wt.%) and RuO_2 were used as the reference catalysts for ORR and OER, respectively. Material characterizations included scanning electron microscopy (SEM), contact angle, x-ray photoelectron spectroscopy (XPS), transmission electron microscopy (TEM), elemental mapping (EDS), N_2 adsorption adsorption/desorption isotherms, and Raman spectroscopy. Electrochemical tests included cyclic voltammetry (CV), linear sweep voltammetry (LSV), electrochemical impedance spectroscopy (EIS), rotating disc electrode (RDE), rotating ring disk electrode (RRDE), accelerated durability test (ADT), chronoamperometry (CA), double layer capacitance (C_{dl}), electrochemically active surface area (ECSA), Faraday efficiency, etc.

1.4. ORR activity Measurements

The ORR performance was tested in 0.1 M KOH solution. The cyclic voltammetry (CV) tests were cycled positively in the potential range from 0 to 1.2 V with a scan rate of 50 mV s^{-1}. Before each CV test, O_2 was bubbled to saturate the electrolyte. The linear sweep voltammetry (LSV) tests were performed at 1600 rpm (50 mV s^{-1}) in the O_2-saturated electrolyte [76]. As previously reported, the Tafel tests were carried out in an O_2-saturated electrolyte at 1600 rpm. Based on the LSV curve, the corresponding Tafel diagram could be drawn [77]. It was determined by the following Tafel equation:.

$$\eta = a + b\log|J| \qquad (1)$$

where η is the overpotential, a is the overpotential value when the current density is a unit value (1A cm^{-2}), b is the Tafel slope, and J is the current density.

RDE measurements were conducted by varying the rotation rates (from 400 to 2500 rpm) in the LSV tests. The Koutecky–Levich (K–L) equation was used to analyze the obtained RDE data, and the transferred electron number (n) was obtained using Equations 2 and 3 [78].

$$\frac{1}{j} = \frac{1}{j_L} + \frac{1}{j_K} = \frac{1}{B\omega^{1/2}} + \frac{1}{nFkC_O} \qquad (2)$$

$$B = 0.62nFC_0D_0^{2/3}\upsilon^{-1/6} \qquad (3)$$

where j represents the measured value of the disk current density; j_L represents the measured value of the diffusion limit current density; j_K represents the measured value of the kinetic-limiting current density; ω is the angular speed of the rotating electrode; F represents the Faraday constant (F = 96485 C mol^{-1}); k is the electron-transfer rate constant; C_O is the oxygen concentration in the test solution (0.1 M KOH, C_O= 1.2 × 10^{-6} mol cm^{-3}); D_O is the oxygen diffusion coefficient in the test solution (0.1 M KOH, D_O= 1.9 × 10^{-5} cm^2 s^{-1}); and υ is the kinematic viscosity of 0.1 M KOH (υ = 0.01 cm^2 s^{-1}).

According to previous reports, the tests for electrochemical impedance spectroscopy (EIS) and chronoamperometry (CA) were conducted at 0.8 V (1600 rpm) in an O_2-saturated 0.1 M KOH electrolyte. Accelerated durability tests (ADT) were carried out to investigate the durability of the catalyst after 5000 cycles of CV tests, and LSV results were compared before and after the ADT. RRDE measurements were also carried out in O_2-saturated 0.1 M KOH electrolyte at a scan rate of 5 mV s^{-1} at 1600 rpm. The hydrogen peroxide yield (H_2O_2 (%)) and electron transfer numbers (n) were obtained from the RRDE voltammograms using the following Equations 4 and 5:

$$n = 4 \times \frac{Id}{Id + (Ir / N)} \qquad (4)$$

$$H_2O_2\ \% = 200 \times \frac{Ir / N}{Id + (Ir / N)} \quad (5)$$

where I_d and I_r are the disk current and the ring current, respectively; N is the H_2O_2 collection efficiency of the Pt ring with the value of 0.37.

1.5. OER activity measurements

OER tests were performed in 0.1 M KOH electrolyte solution. Linear sweep voltammetry (LSV) was carried out at a scan rate of 2 mV s^{-1} for the polarization curves, without iR-compensation correction. The durability of the catalyst after 1000 CV cycles was studied by using the accelerated durability test (ADT). EIS tests were performed at 1.58 V vs. RHE with frequencies ranging from 100 kHz to 100 MHz at a 5mV voltage amplitude. The electrochemically active area was calculated from the electrical double-layer capacitance measurements, which were cycled at scan rates of 40, 60, 80, 100, and 120 mV s^{-1} with a potential range from 0.96 to 1.06 V vs. RHE. Tafel and CA tests were performed using the reported methods [79].

The Faradaic efficiency determination was accepted as an important activity parameter that had to be determined to ensure that the supplied energy was efficiently used for water oxidation as opposed to other side reactions. Equation 6 for calculating FE was performed as follows [80,81]:

$$FE = Ir/(Id \times N) \quad (6)$$

where Ir and Id are the ring current and disk current at a particular potential, respectively; N (0.37) is the current.

1.6. Fabrication of home-made Zn-air batteries

The primary battery was designed at room temperature with an oxygen-saturated 6.0 M KOH solution containing 0.2 M Zn $(Ac)_2$ as the electrolyte, using a polished Zn plate as the anode and the composite substrates (hydrophobic carbon paper + waterproof membrane + nickel foam) produced in Changsha Spring New Energy Technology Co., Ltd. as the catalyst carrier of the air electrode. Catalyst ink was prepared by mixing 50 μL of 5 wt.% Nafion (Aldrich), 1 mL of ethanol, and 50 mg NiCo@NCNTs/HN powder, which was then coated on composite substrates (1 cm×1 cm) as air cathodes. A Celgard 2340 membrane (a 38 um microporous tri-layer membrane with one polyethylene layer between two polypropylene layers) was used as a separator to prevent physical contact between the cathode and anode. The electrocatalyst loading was about 1.0 mg cm^{-2}. The polarization curve was measured at a scan rate of 10 mV s^{-1}. The galvanostatic recharge/discharge cycling measurements were collected on a CT-2001A testing system at a current density of 10 mA cm^{-2}.

2. Results and discussion

Table S1. Chemical compositions (wt. %) of NiCo@NCNTs/HN electrocatalysts obtained from XPS analyses.

Catalysts	C	N	O	Ni	Co
NiCo@NCNTs/HN	50.59	4.55	28.85	0.68	15.33
NiCo/HN-700	83.91	8.69	4.84	0.59	1.97

Table S2. Textural properties of NiCo@NCNTs/HN.

Samples	S_{BET} (m^2 g^{-1})	Pore volume (cm^3 g^{-1})	Average pore width (nm)
NiCo@NCNTs/HN	240	0.276	3.904

Table S3. Textural properties of Ni@NCNTs/HN.

Samples	S_{BET} (m^2 g^{-1})	Pore volume (cm^3 g^{-1})	Average pore width (nm)
Ni@NCNTs/HN	226	0.206	3.404

Table S4. Textural properties of Co@NCNTs/HN.

Samples	S_{BET} (m^2 g^{-1})	Pore volume (cm^3 g^{-1})	Average pore width (nm)
Co@NCNTs/HN	232	0.266	3.764

Table S5. Summary of ORR activities of various catalysts in alkaline electrolyte.

Catalysts	E_0 (V vs. RHE)	$E_{1/2}$ (V vs. RHE)	J_L (mA cm^{-2})	References
NiCo@NCNTs/HN	0.92	0.87	4.96	This work
Ni@NCNTs/HN	0.79	0.73	4.70	This work
Co@NCNTs/HN	0.78	0.72	4.62	This work
NiCo/HN-700	0.72	0.64	4.30	This work
Ag-CeO_2-Co_3O_4/C	-	0.80	6.20	[82]
MnSe@MWCNT	0.94	0.86	6.02	[83]
O-PBA/N-CNT	0.84	0.80	-	[84]
BNF-LCF	0.95	0.81	-	[85]
Mn-Co_3O_4@CNTs	0.99	0.84	5.70	[86]
$FeCo_2O_4$@$FeCo_2S_4$/NF	0.89	0.76	-	[87]
Co_3O_4/B_4C@CoNBC	0.92	0.83	5.00	[88]

Table S6. Summary of OER activities of various catalysts in alkaline electrolyte.

Catalysts	$E_{j=10}$ (V vs. RHE)	Tafel slope	References
NiCo@NCNTs/HN	1.56	66	This work
Ni@NCNTs/HN	1.74	187	This work
Co@NCNTs/HN	1.65	113	This work
NiCo/HN-700	1.64	113	This work
Co-CeO_2/C	1.61	99	[89]
$Ba_{0.5}Sr_{0.5}CoO_{3-\delta}$	1.62	64	[90]
ZnS/NSC-1000	1.62	117	[91]
Co_2P NP	1.59	78	[92]

Table S7. Summary of the ORR/OER bifunctional oxygen electrocatalytic activity of various catalysts in alkaline electrolyte.

Catalysts	$E_{1/2}$ (V vs. RHE)	$E_{j=10}$ (V vs. RHE)	△E (V vs. RHE)	References
NiCo@NCNTs/HN	0.87	1.56	0.69	This work
Co_7Fe_3/CFNC	0.845	1.65	0.81	[93]
Co-N@PCNFs-0.2	0.87	1.66	0.79	[94]
Fe_1Co_3-NC-1100	0.87	1.57	0.70	[95]
LDH@Fe-N-CNFs	0.84	1.58	0.86	[96]
ZGNiFe@NC	0.81	1.55	0.74	[97]
CoO/CoS_2	0.86	1.58	0.72	[98]
Fe@Co-NMC-900	0.78	1.55	0.77	[99]
Fe-Co/Co_3O_4@NC-900	0.84	1.64	0.80	[100]

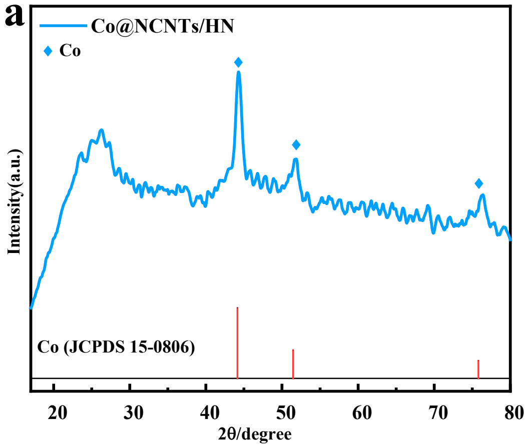

Figure S1. XRD patterns of Co@NCNTs/HN (a) and Ni@NCNTs/HN (b).

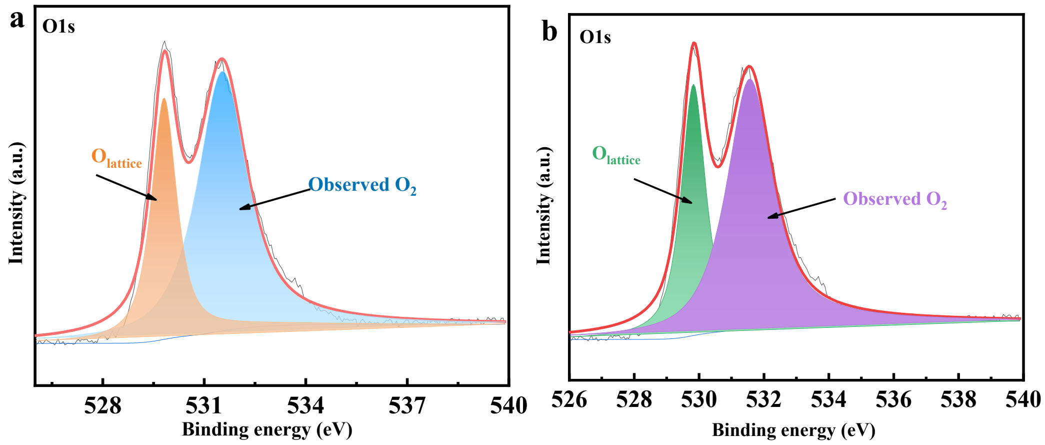

Figure S2. High resolution XPS spectra of O 1s for NiCo@NCNTs/HN (a) and NiCo/HN-700 (b).

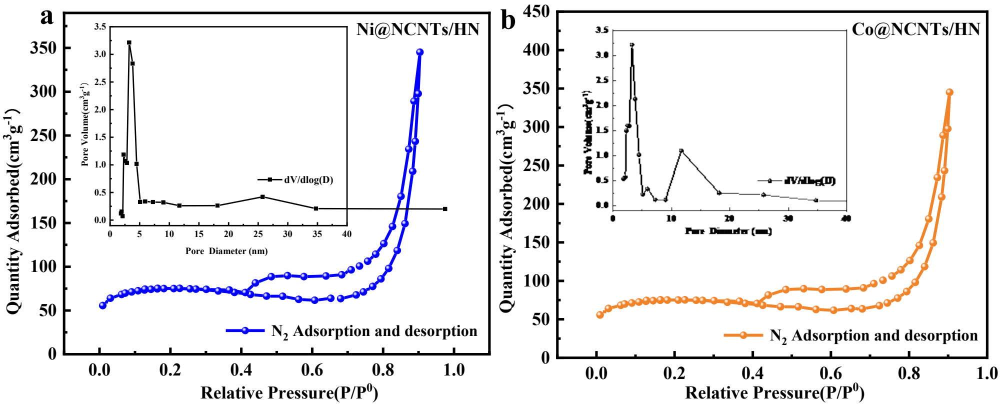

Figure S3. N_2 adsorption/desorption isotherm and pore size distribution (inset) for Ni@NCNTs/HN (a) and Co@NCNTs/HN (b).

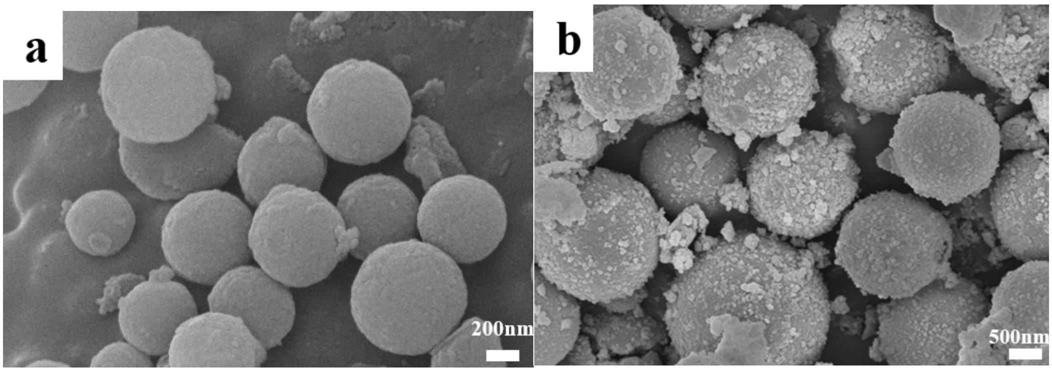

Figure S4. SEM images of Co-MOF (a) and Ni-MOF (b).

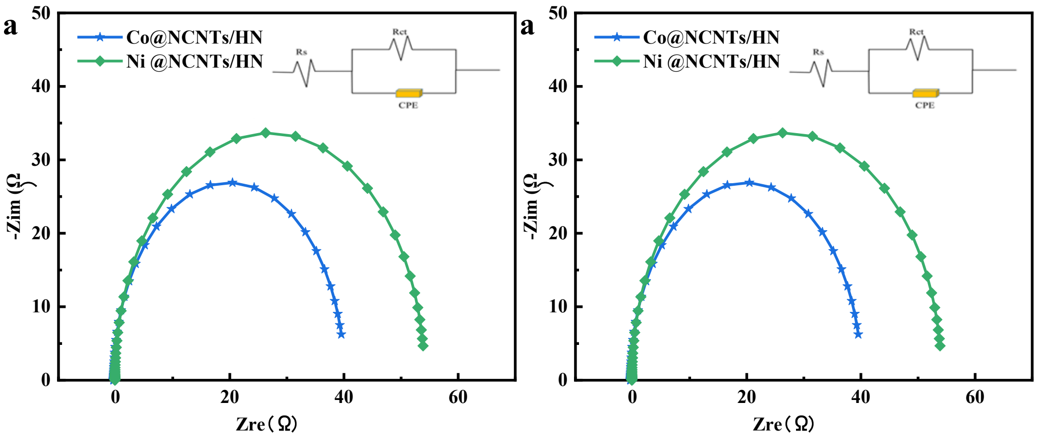

Figure S5. (a) Nyquist curves of Co@NCNTs/HN and Ni@NCNTs/HN in an O_2-saturated 0.1 M KOH solution at an amplitude of 5 mV with a rotation rate of 1600 rpm for ORR; (b) Nyquist curves of Co@NCNTs/HN and Ni@NCNTs/HN in an O_2-saturated 1.0 M KOH solution at an amplitude of 5 mV with a rotation rate of 1600 rpm for OER.

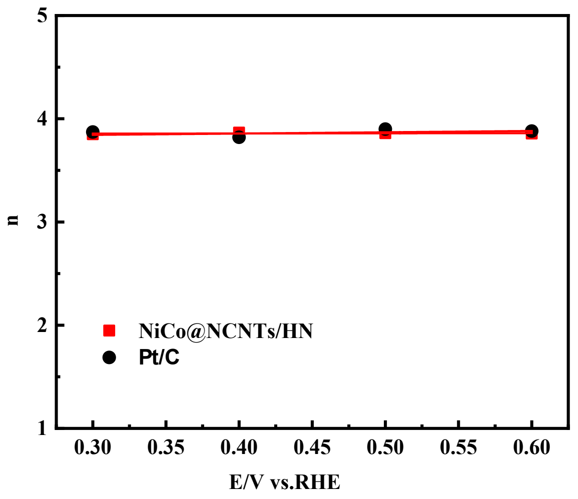

Figure S6. Electron-transfer number n derived from K–L plots at different potentials.

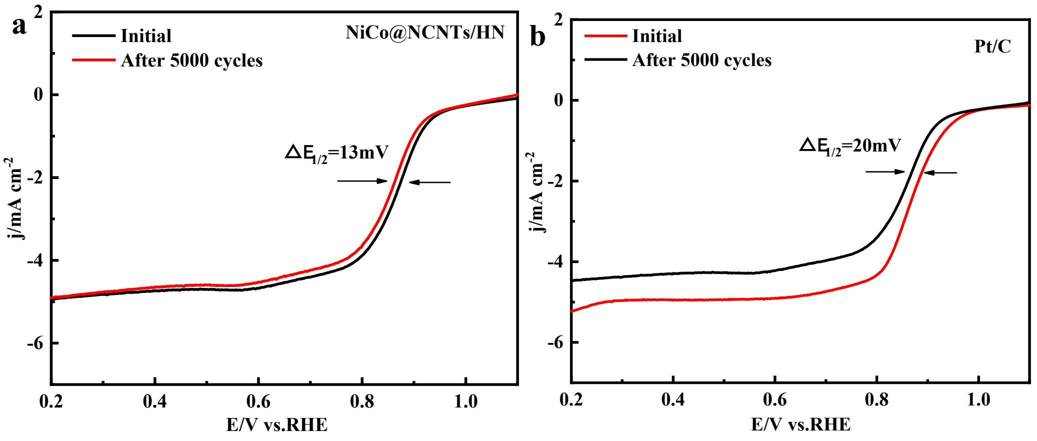

Figure S7. ORR polarization curves of NiCo@NCNTs/HN (a) and Pt/C (b) before and after the continuous CV tests in the O_2-saturated 0.1 M KOH at 1600 rpm.

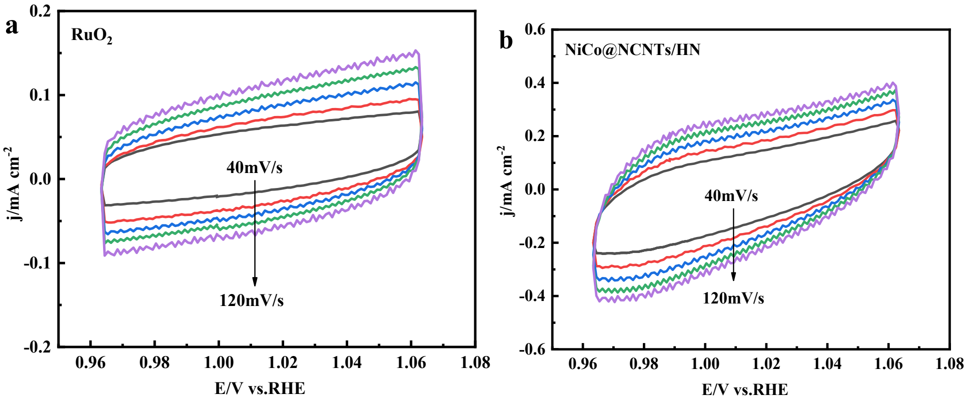

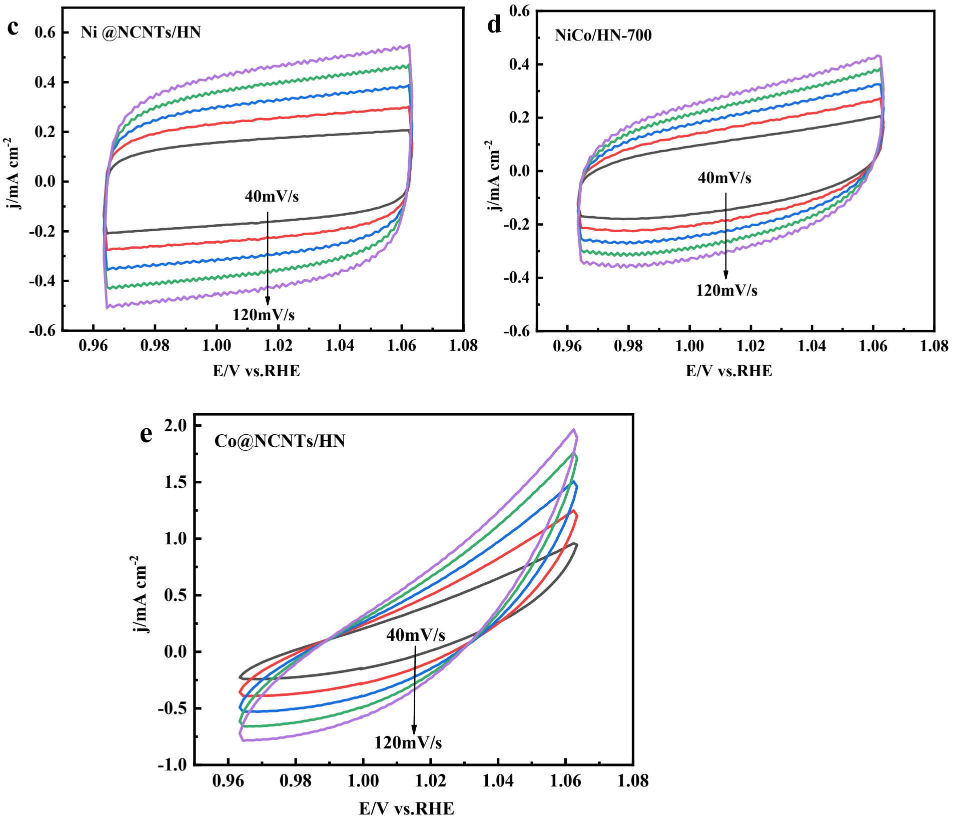

Figure S8. CV curves of RuO_2 (a), NiCo@NCNTs/HN (b), Ni@NCNTs/HN (c), NiCo/HN-700 (d), and Co@NCNTs/HN (e) in 0.1 M KOH electrolyte at different scan rates.

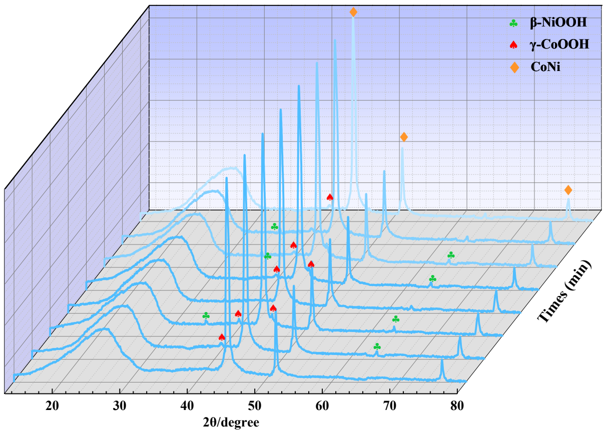

Figure S9. In situ XRD patterns of NiCo@NCNTs/HN collected during OER.

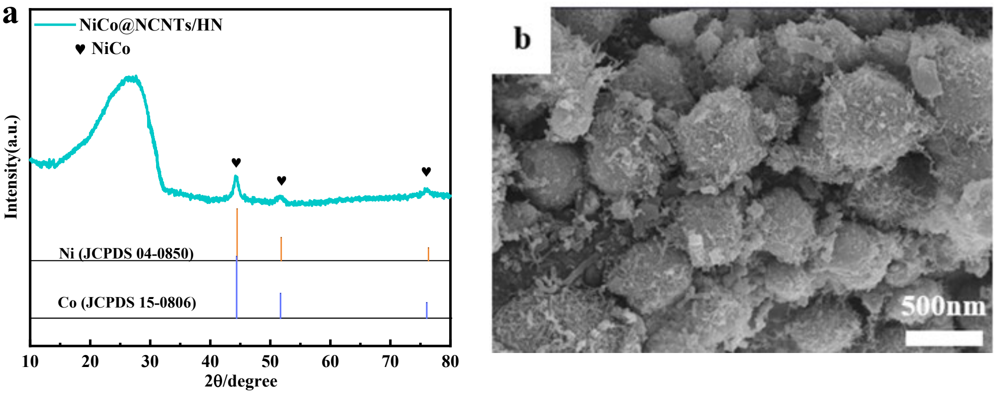

Figure S10. XRD (a) and SEM (b) of the NiCo@NCNTs/HN sample after the chronopotentiometric test for 10 h.

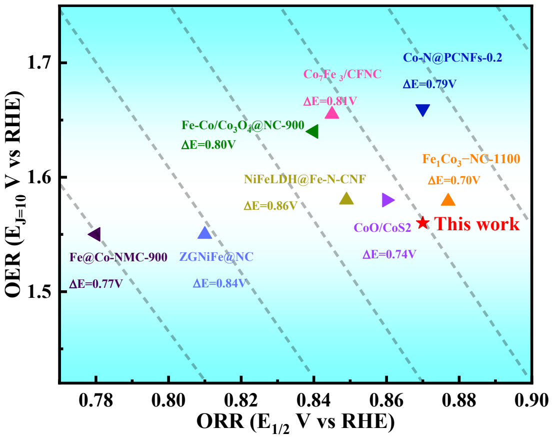

Figure S11. Comparison of OER and ORR activities in this work with representative electrocatalysts in the literature (the dotted lines show the ΔE at constant values).

References

76. Du, J.; You, S.; Li, X.; Tang, B.; Jiang, B.; Yu, Y.; Cai, Z.; Ren, N.; Zou, J. In Situ Crystallization of Active NiOOH/CoOOH Heterostructures with Hydroxide Ion Adsorption Sites on Velutipes-like CoSe/NiSe Nanorods as Catalysts for Oxygen Evolution and Cocatalysts for Methanol Oxidation. *ACS Appl Mater Interfaces* **2020,** *12*, 686-697. 10.1021/acsami.9b16626
77. Guo Y.; Yuan P.; Zhang J.; Xia H.; Cheng F.; Zhou M.; Li J.; Qiao Y.; Mu S.; Xu Q. Co_2P-CoN double active centers confined in N-doped carbon nanotube: heterostructural engineering for trifunctional catalysis toward HER, ORR, OER, and Zn-air batteries driven water splitting. *Adv. Funct. Mater.* **2018**, 28, 1805641. 10.1002/adfm.201805641
78. Jian H.; Gu J.; Zheng X.; Liu M.; Qiu X.; Wang L.; Li W.; Chen Z.; Ji X.; Li J. Defect-rich and ultrathin N doped carbon nanosheets as advanced trifunctional metal-free electrocatalysts for the ORR, OER and HER. *Energy Environ. Sci.* **2019**, 12, 322-333. 10.1039/c8ee03276a
79. Anantharaj S.; Karthik P.; Kundu S. Self-assembled IrO_2 nanoparticles on a DNA scaffold with enhanced catalytic and oxygen evolution reaction (OER) activities. *J. Mater. Chem. A* **2015**, 3, 24463-24478. 10.1039/c5ta07075a
80. Guo, Y.; Yuan, P.; Zhang, J.; Xia, H.; Cheng, F.; Zhou, M.; Li, J.; Qiao, Y.; Mu, S.; Xu, Q. Co_2P-CoN Double Active Centers Confined in N-Doped Carbon Nanotube: Heterostructural Engineering for Trifunctional Catalysis toward HER, ORR, OER, and Zn-Air Batteries Driven Water Splitting. *Advanced Functional Materials* **2018,** *28*. 10.1002/adfm.201805641
81. Wang, R.; Liu, B.; You, S.; Li, Y.; Zhang, Y.; Wang, D.; Tang, B.; Sun, Y.; Zou, J. Three-dimensional Ni_3Se_4 flowers integrated with ultrathin carbon layer with strong electronic interactions for boosting oxygen reduction/evolution reactions. *Chemical Engineering Journal* **2022,** *430*. 10.1016/j.cej.2021.132720
82. Li, T.; He, Z.; Liu, X.; Jiang, M.; Liao, Q.; Ding, R.; Liu, S.; Zhao, C.; Guo, W.; Zhang, S.; He, H. Interface interaction of Ag-CeO_2-Co_3O_4 facilitate ORR/OER activity for Zn-air battery. *Surfaces and Interfaces* **2022,** *33*. 10.1016/j.surfin.2022.102270

83. Singh, H.; Marley-Hines, M.; Chakravarty, S.; Nath, M., Multi-walled carbon nanotube supported manganese selenide as a highly active bifunctional OER and ORR electrocatalyst. *Journal of Materials Chemistry A* **2022,** *10,* 6772-6784. 10.1039/D1TA09864K
84. Aulia, S.; Lin, Y.; Chang, L.; Wang, Y.; Lin, M.; Ho, K.; Yeh, M. Oxygen Plasma-Activated NiFe Prussian Blue Analogues Interconnected N-Doped Carbon Nanotubes as a Bifunctional Electrocatalyst for a Rechargeable Zinc–Air Battery. *ACS Applied Energy Materials* **2022,** *5,* 9801-9810. 10.1021/acsaem.2c01475
85. Wang, Y. ;Gan, R. ;Zhao, S.; Ma, W.; Zhang, X.; Song, Y.; Ma, C. ; Shi, J. B, N, F tri-doped lignin-derived carbon nanofibers as an efficient metal-free bifunctional electrocatalyst for ORR and OER in rechargeable liquid/solid-state Zn-air batteries, *Appl. Surf. Sci.* **2022**,598, 153891. 10.1016/j.apsusc.2022.153891.
86. Zhang, X.; Liu, Q.; Liu, S.; Wang, E. Manganese-doped cobalt spinel oxide as bifunctional oxygen electrocatalyst toward high-stable rechargeable Zn-air battery, *ElectrochimicaActa.* **2023,**437,141477. 10.1016/j.electacta.2022.141477.
87. Yang, T.; Hu, X.; Zheng, W.; Li, Z.; Wu, D.; Lu, G.; Zhao, Q.; Yang, Z.; Wang, R.; Xu, C., Rational Design of an FeCo2O4@FeCo2S4 Heterostructure as an Efficient Bifunctional Electrocatalyst for Zn–Air Batteries. *ACS Applied Energy Materials* **2022,** *5,* 9742-9749. 10.1021/acsaem.2c01417
88. Liu, X.; Zang, J.; Chelimuge; Song, S.; Gao, H.; Zhou, S.; Wang, Y. A hybrid of Co_3O_4 nanoparticles coupled with B, Co/N-codoped C@B_4C as an efficient bifunctional catalyst for oxygen reduction and oxygen evolution reactions. *International Journal of Hydrogen Energy* **2023,** *48* (2), 542-552. 10.1016/ j.ijhydene
89. Liu, Z.; Wan, J.; Li, M.; Shi, Z.; Liu, J.; Tang, Y. Synthesis of Co/CeO_2/C hetero-particles with abundant oxygen-vacancies supported by carbon aerogels for ORR and OER. *Nanoscale* **2022,** *14,* 1997-2003. 10.1039/D1NR07595K
90. Mondal, R.; Ratnawat, H.; Mukherjee, S.; Gupta, A.; Singh, P. Investigation of the Role of Sr and Development of Superior Sr-Doped Hexagonal BaCoO3−δ Perovskite Bifunctional OER/ORR Catalysts in Alkaline Media. *Energy & Fuels* **2022,** *36* (6), 3219-3228. 10.1021/acs.energyfuels.2c00357
91. Peng, Y.; Zhang, F.; Zhang, Y.; Luo, X.; Chen, L.; Shi, Y. ZnS modified N, S dual-doped interconnected porous carbon derived from dye sludge waste as high-efficient ORR/OER catalyst for rechargeable zinc-air battery. *J Colloid Interface Sci* **2022,** *616,* 659-667. 10.1016/j.jcis.2022.02.102
92. Jebaslinhepzybai, B. T.; Partheeban, T.; Gavali, D. S.; Thapa, R.; Sasidharan, M., One-pot solvothermal synthesis of Co_2P nanoparticles: An efficient HER and OER electrocatalysts. *International Journal of Hydrogen Energy* **2021,** *46,* 21924-21938. 10.1016/j.ijhydene.2021.04.022
93. Tu, T.; Zhou, X.; Zhang, P.; Tan, L.; Xu, Z.; Liu, M.; Li, W.; Kang, X.; Wu, Y.; Zheng, J. Co_7Fe_3 Nanoparticles Confined in N-Doped Carbon Nanocubes for Highly Efficient, Rechargeable Zinc–Air Batteries. *ACS Sustainable Chemistry & Engineering* **2022,** *10,* 8694-8703. 10.1021/acssuschemeng.1c08618
94. Gao, K.; Shen, M.; Duan, C.; Xiong, C.; Dai, L.; Zhao, W.; Lu, W.; Ding, S.; Ni, Y., Co-N-Doped Directional Multichannel PAN/CA-Based Electrospun Carbon Nanofibers as High-Efficiency Bifunctional Oxygen Electrocatalysts for Zn–Air Batteries. *ACS Sustainable Chemistry & Engineering* **2021,** *9,* 17068-17077. 10.1021/acssuschemeng.1c06040
95. He, Y.; Yang, X.; Li, Y.; Liu, L.; Guo, S.; Shu, C.; Liu, F.; Liu, Y.; Tan, Q.; Wu, G. Atomically Dispersed Fe–Co Dual Metal Sites as Bifunctional Oxygen Electrocatalysts for Rechargeable and Flexible Zn–Air Batteries. *ACS Catalysis* **2022,** *12,* 1216-1227. 10.1021/acscatal.1c04550
96. Wu, D.; Hu, X.; Yang, Z.; Yang, T.; Wen, J.; Lu, G.; Zhao, Q.; Li, Z.; Jiang, X.; Xu, C. NiFe LDH Anchoring on Fe/N-Doped Carbon Nanofibers as a Bifunctional Electrocatalyst for Rechargeable Zinc–Air Batteries. *Industrial & Engineering Chemistry Research* **2022,** *61,* 7523-7528. 10.1021/acs.iecr.1c04694
97. Li, W.; Wu, Y.; Chen, M.; Dai, P.; Jiang, T.; Zhou, S. Ultrathin nitrogen-doped defective carbon layer embedded with NiFe for solid zinc-air batteries. *Journal of Alloys and Compounds* **2022,** *925.* 10.1016/j.jallcom.2022.166658
98. Qin, T.; Ding, Y.; Zhang, R.; Gao, X.; Tang, Z.; Liu, Y.; Gao, D. Bifunctional CoO/CoS_2 hierarchical nanospheres electrocatalyst for rechargeable Zn-Air battery. *FlatChem* **2022,** *32.* 10.1016/j.flatc.2022.100343
99. Zhang, Y.; li, Y.; Shi, K.; Zhu, Z.; Li, X.; Xu, H.; Gao, J. Bimetallic dispersion zeolitic imidazolate framework derived spherical porous bifunctional catalysts for liquid/solid Zn-Air batteries. *Journal of Alloys and Compounds* **2022,** *925.* 10.1016/j.jallcom.2022.166680
100. Kong, Q.; Lv, X.; Weng, C.; Ren, J.; Tian, W.; Yuan, Z. Curving Engineering of Hollow Concave-Shaped Rhombic Dodecahedrons of N-Doped Carbon Encapsulated with Fe-Doped Co/Co_3O_4 Nanoparticles for an Efficient Oxygen Reduction Reaction and Zn–Air Batteries. *ACS Sustainable Chemistry & Engineering* **2022,** *10,* 11441-11450. 10.1021/acssuschemeng.2c03952
